# HNRNPA2B1-mediated m6A modification of TLR4 mRNA promotes progression of multiple myeloma

**DOI:** 10.1186/s12967-022-03750-8

**Published:** 2022-11-18

**Authors:** Lina Quan, Chuiming Jia, Yiwei Guo, Yao Chen, Xinya Wang, Qiuting Xu, Yu Zhang

**Affiliations:** 1 Hematology Department, Harbin Medical University Cancer Hospital, Harbin, Heilongjiang People’s Republic of China; 2 Immunology Department, Harbin Medical University, Harbin, Heilongjiang People’s Republic of China

**Keywords:** HNRNPA2B1, TLR4, Multiple myeloma, N6-methyladenosine methylation

## Abstract

**Background:**

Multiple myeloma (MM) is a malignancy of plasma cells that remains incurable. Toll-like receptor 4 (TLR4) acts as a stress-responsive signal, protecting mitochondria during proteasome inhibitor (PI) exposure, maintaining mitochondrial metabolism and increasing drug resistance in MM. However, the mechanism of TLR4 regulation remains elusive.

**Aims:**

The purpose of this study was to investigate the methylation pattern of multiple myeloma and its effect on the expression of HNRNPA2B1 and downstream targets.

**Methods:**

The methylation level in MM and normal bone marrow specimens was detected using a colorimetric assay. HNRNPA2B1 gene knockdown was achieved in RPMI 8226 MM cells via adenovirus transfection. CCK8 and flow cytometric assays were used to detect proliferation and apoptosis, respectively. Transcriptome sequencing and m6A methylation MeRIP sequencing were applied, and differentially expressed genes (DEGs) were detected. Three independent NCBI GEO datasets were applied to examine the effects of HNRNPA2B1 and TLR4 expression on MM patient survival.

**Results:**

HNRNPA2B1 promoted MM progression. Clinical data from database revealed that HNRNPA2B1 was adverse prognostic factor for survival among MM patients. Furthermore, transcriptome sequencing and methylation sequencing showed that HNRNPA2B1 recognized and was enriched at the m6A sites of TLR4 and TLR4 was down-regulated of both the m6A level and transcription level in HNRNPA2B1-knockdown MM cells. Moreover, TLR4 was an adverse survival prognostic factor based on database analysis.

**Conclusion:**

Overall, our study implies that the RNA-binding protein HNRNPA2B1 increases cell proliferation and deregulates cell apoptosis in MM through TLR4 signaling. Our study suggests HNRNPA2B1 as a potential therapeutic target for MM.

**Supplementary Information:**

The online version contains supplementary material available at 10.1186/s12967-022-03750-8.

## Introduction

Multiple myeloma (MM) is a clonal B-cell malignancy that accounts for approximately 10% of all hematologic malignancies and is characterized by abnormal plasma cell proliferation and abnormal globulin secretion in bone marrow [1]. Although survival outcomes in MM patients have significantly improved over the past 20 years, the disease remains incurable [2, 3]. Additionally, chemotherapy resistance develops often in MM patients during treatment, stemming from a variety of factors including genetic abnormalities [4]. Therefore, new therapeutic targets are urgently needed. Transcriptomic studies have provided important information on disease-related pathways and critical genes in addition to revealing new targets for the treatment of diseases [5–7].

N6-methyladenosine (m6A) methylation is the most common post-transcriptional modification found on eukaryotic mRNA so far and also widely exists in a variety of bacteria and RNA viruses [8]. m6A modification participates in the regulation of mRNA translation, splicing processing, as well as nuclear transport and degradation, which determines the entire life process of mRNA [9]. Over the past few decades, the biological functions regulated by m6A have been shown to be involved in several types of tumor progression [10]. However, the critical role of m6A modification in MM is still elusive, and the regulatory mechanisms of MM in general are not fully understood.

Heterogeneous nuclear ribonucleoprotein A2B1 (HNRNPA2B1) is one of the main m6A readers [11, 12]. The reader is a methylated reading protein that recognizes RNA methylation modification and performs its functions by specifically binding to the m6A-modified region or altering the RNA secondary structure to facilitate binding to RNA [13]. Recently, abnormal HNRNPA2B1 expression has been discovered in various disease states and linked closely to tumor progression. HNRNPA2B1 may play key roles in cancer development due to its ability to accelerate pre-mRNA processing through the function of RNA binding [14].

In the present study, we discovered the hypermethylated status of MM and the high expression of HNRNPA2B1 in MM. Through transcriptome and m6A sequencing, we identified Toll-like receptor 4 (TLR4) as a target of HNRNPA2B1-mediated m6A modification. HNRNPA2B1 knockdown decreased the TLR4 mRNA level and simultaneously abolished TLR4 mRNA m6A modification. Through analysis of gene expression omnibus (GEO) datasets, HNRNPA2B1 and TLR4 were found to be adverse prognostic factors for survival among MM patients. Our results reveal the biological role of HNRNPA2B1 in mediating m6A modification in MM, indicating that HNRNPA2B1 plays an important role in MM pathogenesis via epigenetic regulation a critical target TLR4. From these findings, we propose that HNRNPA2B1 may be a novel therapeutic target for evaluating MM progression under m6A-based post-transcriptional regulation through TLR4 pathway.

## Materials and methods

### MM specimens and cell lines

Bone marrow specimens were obtained from 15 newly diagnosed MM patients and 15 normal controls (NC) at Harbin Medical University Cancer Hospital, China, between January 2019 and December 2020. The National Comprehensive Cancer Network clinical practice guidelines were used to diagnose MM.

The MM cell line, RPMI 8226 was purchased from the Cell Bank of the Chinese Academy of Sciences (Shanghai, China). The cells were maintained in RPMI 1640 medium (Gibco, Grand Island, NY, USA) supplemented with 10% (for RPMI 8226) fetal bovine serum (FBS, Hyclone, Logan, UT, USA) and 1% penicillin–streptomycin at 37 ℃ in a humidified incubator containing 5% CO_2_.

### RNA extraction, reverse transcription (RT) and quantitative PCR (qPCR)

Total RNA was isolated using Trizol Reagent (Invitrogen, USA). cDNA was synthesized from 1 µg of total RNA using the Prime Script™ RT reagent kit (Takara Bio, Inc., Otsu, Japan). Relative gene expression was assessed by real-time RT-PCR using the SYBR Premix Ex Taq TM kit (Takara Bio, Inc., Otsu, Japan) on the ABI 7900HT Real-Time PCR system (Applied Biosystems Life Technologies, Foster City, CA, USA) according to the manufacturers’ instructions and suggested protocol. The primers used for qRT-PCR are listed in Additional file 1: Table S1. Each analysis was carried out in triplicate.

### Cell proliferation and apoptosis assays

Aliquots of 3 × 10^4^ cells were suspended in 100 µl RPMI 1640 medium and then seeded into 96-well plates. The CCK8 (Dojindo Molecular Technologies, Japan) assay was conducted according to the manufacturer’s instructions. Briefly, after cell culture, 10 µl CCK8 solution was added into each 96-well, and after incubation for 2 h, the absorbance at 450 nm wavelength was measured. Each analysis was carried out in triplicate.

Apoptosis was evaluated by Annexin V-APC/propidium iodide (PI) staining. Briefly, cells were resuspended in binding buffer, and then Annexin V-APC and PI were added to each sample for 15 min and incubation at room temperature in darkness. Flow cytometry CantoII (BD Biosciences, San Jose, CA, USA) was used to evaluate the numbers of apoptotic cells. Each analysis was carried out in triplicate.

### Adenovirus vector plasmid for transfection of RPMI 8226 cells

Adenovirus expressing HNRNPA2B1-shRNAs was purchased from Hanheng Bioscience Co., Ltd. (Shanghai, China). The HNRNPA2B1 shRNA sequences were: sh1,5′-TCGAGGCCATGGCTGCAAGACCTCATTCAATTTCAAGAGAATGAATGAGGTCTTGCAGCCATGGTTTTTTA-3′; sh2, 5′-AGCTTAAAAAACCATGGCTGCAAGACCTCATTCAATTCTCTTGAAATTGAATGAGGTCTTGCAGCCATGGCC-3′; and sh3, 5′-TCGAGGAGGAACAGTTCCGTAAGCTCTTTATTTCAAGAGAATAAAGAGCTTACGGAACTGTTCCTTTTTTTA-3′. Aliquots of 5 × 10^6^ cells were plated onto 25-cm^2^ plates for 48 h before transfection with Ad-eGFP or Ad-HNRNPA2B1 with RPMI 1640 medium for 4 h at 37 °C. The cells were then washed twice with RPMI 1640 medium and once with RPMI 1640 medium containing 10% FBS, and then incubated for 2 days at 37 °C. After the formation of a stable cell line, RT-qPCR and western blot analyses were conducted to screen the RPMI 8226 with HNRNPA2B1 knockdown.

### RNA m6A quantification

After extraction, the quality and quantity of the total RNA were measured by the NanoDrop (Thermo Fisher Scientific, Waltham, MA, USA). The EpiQuik m6A RNA Methylation Quantification Kit was then used to examine the m6A modification level according to the manufacturer’s instructions. Briefly, RNA was coated onto the assay wells, which were then washed and incubated with capture antibody. After antibody addition, the m6A level was detected by measurement of the absorbance at 450 nm (optical density [OD]450). Each analysis was carried out in triplicate.

### RNA-sequencing

To assess the RNA degradation and contamination, 1% agarose gel electrophoresis was conducted, and the NanoPhotometer® spectrophotometer (IMPLEN, CA, USA) was used to test the RNA purity. RNA integrity was assessed by using the Bioanalyzer 2100 System (Agilent Technologies, CA, USA).

For RNA-sequencing of 13 paired samples, mRNA Capture Beads with Oligo(dT) (VAHTS, Nanjing, China) were first used to capture mRNA. mRNA was then purified with binding and washing buffer. mRNA was then randomly fragmented and reverse transcribed into cDNA in the corresponding buffer. Random primers were used to synthesize the first cDNA strand, and dNTPs/DNA polymerase I (ABclonal, MA, USA) was used to synthesize the second cDNA strand. After dA—tailing of 3' DNA fragments, UMI (Novogene, Beijing, China) and sequence adapter were used to ligate DNA. After PCR, the product was purified and collected using DNA Clean Beads (Beckman Coulter, CA, USA) and nuclease-free H_2_O. Each analysis was carried out in triplicate. After quality assessment, the prepared library was sequenced on the Illumina platform (Illumina Novaseq, Inc., USA).

### M6A-RNA immunoprecipitation (MeRIP) assay and m6A sequencing

The mRNA m6A of 15 paired samples was sequenced by MeRIP-seq at Novogene (Beijing, China). The degradation and contamination of the extracted RNA were tested as described above. For immunoprecipitation, fragmented mRNA (~ 100 nt) was incubated with anti-m6A antibody at 4 ℃ for 2 h (Synaptic Systems). m6A modified-mRNA was then enriched and detected through qRT-PCR or next generation sequencing using Illumina HiSeq 2000 (Illumina Inc.). Each analysis was carried out in triplicate. The NEBNext ultraRNA library prepare kit for Illumina (Illumina, Inc., USA) was used for library construction. Finally, the library preparation was sequenced by an Illumina Novaseq platform.

### Dot blot for measuring m6A

Nitrocellulose membrane was fixed in plates. Then 10 µg RNA per well was added to the dot bolt apparatus. Then RNA was crosslinked to the membrane using an ultraviolet (UV) crosslinker. The membrane was then washed with washing buffer and blocked with blocking buffer at room temperature for 30 min. After that, the membrane was incubated with anti-m6A antibody at 4 °C. Then the membrane was washed, incubated with western blotting reagent, and exposed to autoradiography.

### NCBI GEO microarray dataset analysis

GEO datasets GSE116294, GSE80608 and GSE141260 were used to compare the expression of HNRNPA2B1 at different stage of monoclonal gammopathy. GSE116294 consisted of 69 clinical samples, including 4 normal samples, 50 MM samples at diagnosis and 15 plasma cell leukemia (PCL) samples at diagnosis. GSE80608 contained 10 control, 10 monoclonal gammopathy of undetermined significance (MGUS) and 10 MM samples. GSE141260 contained 10 control and 10 MM samples. Additionally, GSE4202, GSE24080 and GSE136400 were used to estimate the relationship between the expression of HNRNPA2B1/TLR4 and the disease prognosis of MM.

Based on the Affymetrix-GPL570 platform, the data for gene expression and survival were obtained from the GEO datasets GSE4204 (newly diagnosed MM, n = 538) and GSE24080 (newly diagnosed MM, n = 559). In GSE4204, the samples were divided into two subgroups based on the median gene expression values, the survival curves were drawn to analyze overall survival (OS) between different expression subgroups. In GSE24080, the clinical endpoints were OS and event-free survival (EFS) at 24 months, and we compared the expression levels of hnRNPA2B1 and TLR4 between two subgroups with different survival outcomes.

In addition, GSE136400 dataset was used to analyze the relationship between TLR4 expression and survival of MM patients. The dataset consisted of 1424 samples from different times, including before treatment, post-treatment but pre-transplantation, post-transplantation, pre-consolidation, etc., and we selected all samples before treatment (n = 354) for further analysis. The gene expression data were analyzed using the GPL27143 platform Affymetrix Human Genome U133 Plus 2.0 Array. X-tile software (v3.6.1, Yale University, New Haven, CT, USA) was used to confirm the cut-off value of TLR4 expression, and the patients were divided into two subgroups: TLR4high and TLR4low. Survival curves were drawn to analyze OS and progression-free survival (PFS) between the two subgroups.

### Western blot analysis

After collection of cells and tissues, radioimmunoprecipitation assay buffer (Beyotime Institute of Biotechnology, Shanghai, China) was added into the pellet to extract proteins. After extraction, concentration of total protein was measured using a BCA protein assay kit (Beyotime Institute of Biotechnology). Agarose gel electrophoresis was performed after the addition of 2X sodium dodecyl sulfate loading buffer and incubated at 100 °C for 5 min. After transfer onto polyvinylidene difluoride membranes and blocking using 5% milk, the membranes were incubated with primary antibodies against TLR4 (1:500; Affinity Biosciences, Jiangsu, China), HNRNPA2B1 (1:500; Affinity Biosciences) and GAPDH (1:500; Affinity Biosciences) overnight at 4 °C. Then the secondary antibodies were applied to the membranes. After a series of washing steps, the membranes were visualized using chemiluminescence (Beyotime Institute of Biotechnology). Each analysis was carried out in triplicate.

### Statistical analysis

Statistical analyses were conducted by GraphPad Prism 8 software (GraphPad Software, Inc., San Diego, CA, USA). Survival curves were drawn by the Kaplan–Meier approach, and the log-rank test was used for comparisons between groups. The differences between two groups were analyzed by unpaired t tests, and differences between multiple groups were analyzed by one-way analysis of variance (ANOVA). In all statistical analyses, *p*-values < 0.05 were considered statistically significant.

## Results

### M6A reader HNRNPA2B1 is highly expressed in MM

**Fig. 1:**
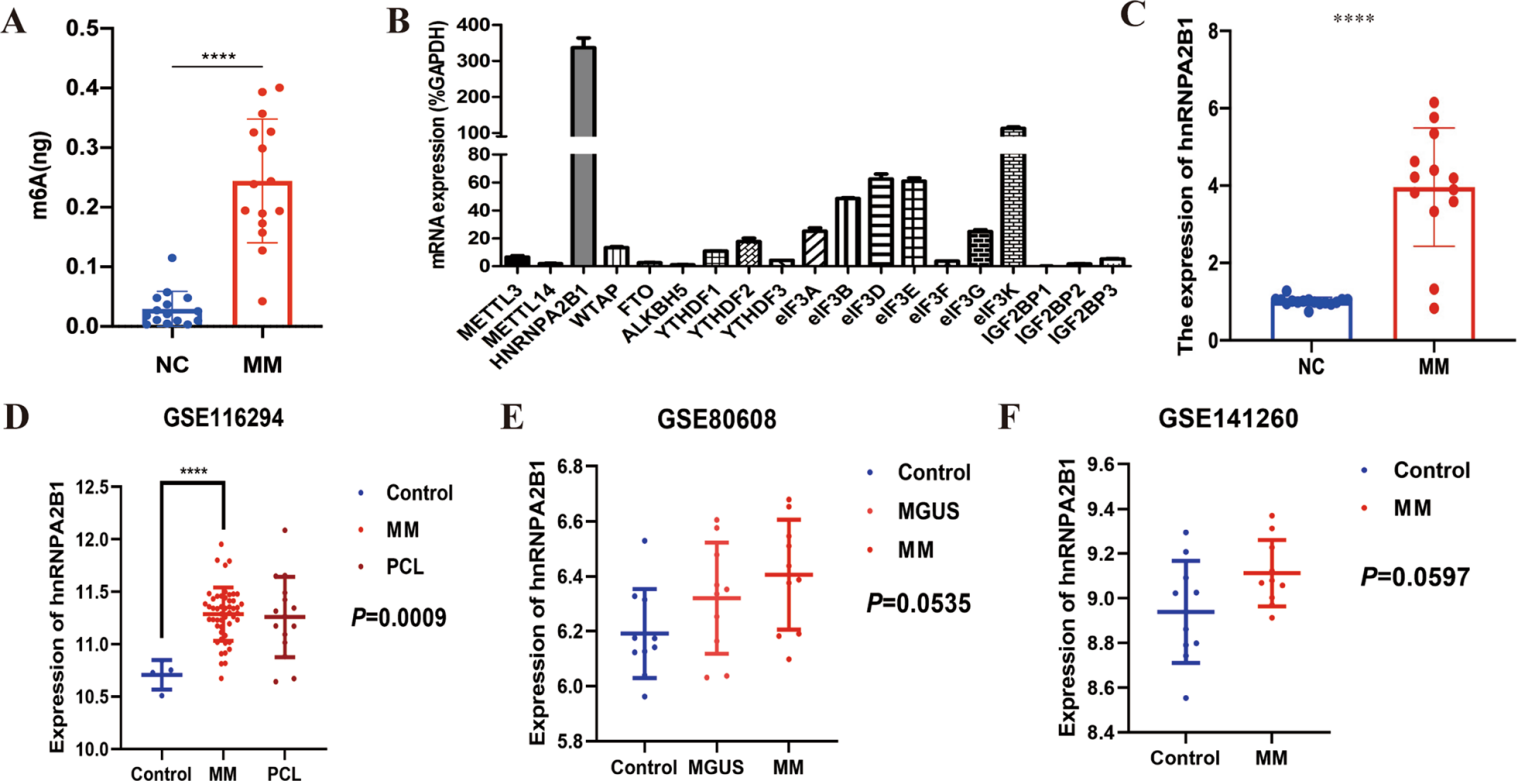
Detection of m6A methylation level in MM. **A** m6A methylation levels in bone marrow from normal controls (n = 15) and MM patients (n = 15) were analyzed via dot blot assay. **B** Total RNA was extracted from the MM cell line RPMI 8226 for qRT-PCR of “writer”, “eraser” and “reader” genes. **C** Bone marrow specimens from 13 newly diagnosed MM patients and 13 normal controls were used for RNA extraction followed by qRT-PCR detection of HNRNPA2B1 expression. **D**–**F** The expression of HNRNPA2B1 were higher in MM than the counterpart in normal control

To investigate the role of m6A modification in MM, we first conducted a dot blot assay to examine m6A methylation levels in bone marrow specimens from MM patients and normal controls. The m6A methylation level in MM bone marrow was significantly higher than that in the normal control bone marrow (*p* < 0.0001), with overall m6A average values for 15 MM patients and 15 normal controls of 0.246 ng and 0.029 ng, respectively (Fig. 1A).

As m6A is regulated by the multiple “writer”, “eraser” and “reader” genes, including *METTL3, METTL14, HNRNPA2B1, WTAP, FTO, ALKBH5, YTHDF1/2/3, IGF2BP1/2/3, eIF3* and others [10]_,_ we next explored dysregulation of these genes in MM. qRT-PCR was performed to explore the expression of these genes in the RPMI 8226 MM cell line. The methylation “reader” gene HNRNPA2B1 was highly expressed in RPMI 8226 cells (Fig. 1B). Furthermore, the expression level of HNRNPA2B1 in bone marrow was significantly higher in 13 newly diagnosed patients with MM than in 13 normal controls (Fig. 1C).

In addition to MM cell line RPMI 8226 and bone marrow specimen, the elevated expression of HNRNPA2B1 in MM was also authenticated in GEO dataset. In GSE116294, the expression of HNRNPA2B1 did not differ among normal control, MM and PCL (*P* = 0.0795). However, as shown in Fig. 1D, the expression level of HNRNPA2B1 in MM patients was significantly higher compared with that of control patients (*P* = 0.0009). In GSE80608 and GSE141260, the expression of HNRNPA2B1 appeared to be higher in the MM group than in the normal group, but the difference did not reach statistical significance (Fig. 1E and F). Together, these results indicate that HNRNPA2B1 expression and m6A methylation level were upregulated in MM.

### HNRNPA2B1 Knockdown decreases proliferation and increases apoptosis among MM cells

**Fig. 2:**
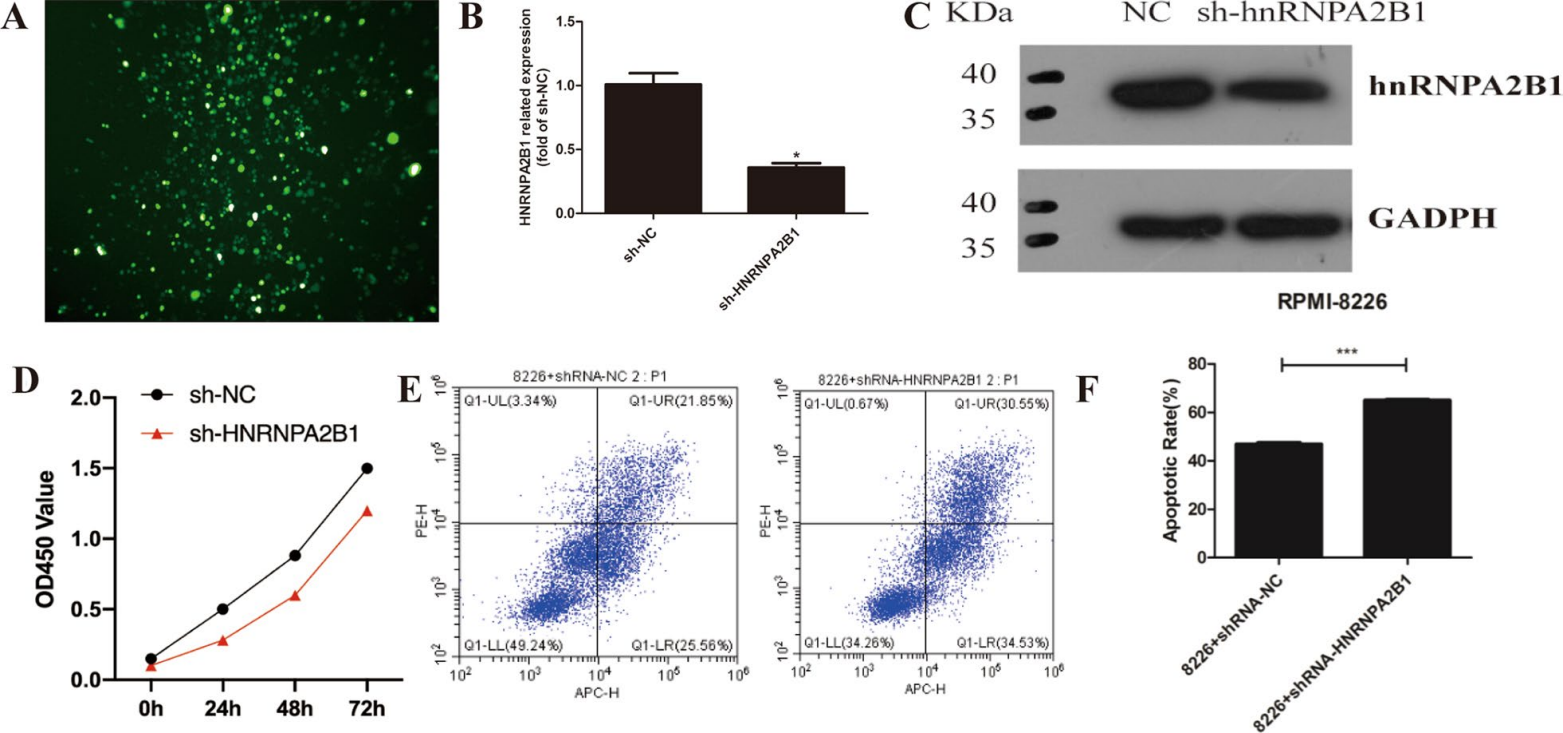
Adenovirus vector plasmid-mediated knockdown of HNRNPA2B1 in transfected RPMI 8226 cells and corresponding effects on cell proliferation and apoptosis. **A** Expression efficiency of GFP/RFP fluorescence of adenovirus vector plasmid in transfected RPMI 8226 cells after 72 h. Multiplicity of infection (MOI) refers to the number of virus particles infecting each cell. **B** qRT-PCR showed HNRNPA2B1 expression was significantly down-regulated at 72 h after transfection of RPMI 8226 cells with the adenoviral vector plasmid. **C** Western blotting showed HNRNPA2B1 protein expression was significantly down-regulated at 72 h after RPMI 8226 cells with the adenoviral vector plasmid. **D** CCK8 assay showing reduced proliferation of RPMI 8226 cells with HNRNPA2B1 knockdown compared with control cells. **E** Flow cytometric detection of apoptosis based on Annexin-V and PI staining in RPMI 8226 cells with HNRNPA2B1 knockdown versus control cells. Diagrams Q-UL, UR, LR, and LL represent necrotic, late apoptotic, early apoptotic, and live cells, respectively. **F** Statistical results from the apoptosis assay in E

The sharp increase in HNRNPA2B1 expression in MM indicates its potential role in myeloma process. Thus, we investigated its influence on cell proliferation and apoptosis in MM. First, RPMI 8226 myeloma cells were transfected with the constructed HNRNPA2B1 viral plasmid (sh-HNRNPA2B1-8226, Fig. 2A), and the transfection efficiency was verified by qPCR. The expression of HNRNPA2B1 in the transfected RPMI 8226 cells was reduced by more than 50%, and the protein level of HNRNPA2B1 in these cells also was reduced (Fig. 2B and C). CCK8 assay results indicated that MM cell proliferation was significantly reduced at 24, 28, and 72 h after HNRNPA2B1 knockdown in a time-dependent manner (Fig. 2D). Additionally, flow cytometric detection of apoptotic MM cells showed that apoptosis among these cells was increased significantly at 72 h after HNRNPA2B1 knockdown, with the greatest increase in late apoptosis (Fig. 2E and F).

### HNRNPA2B1 plays multiple roles in myeloma cells

**Fig. 3:**
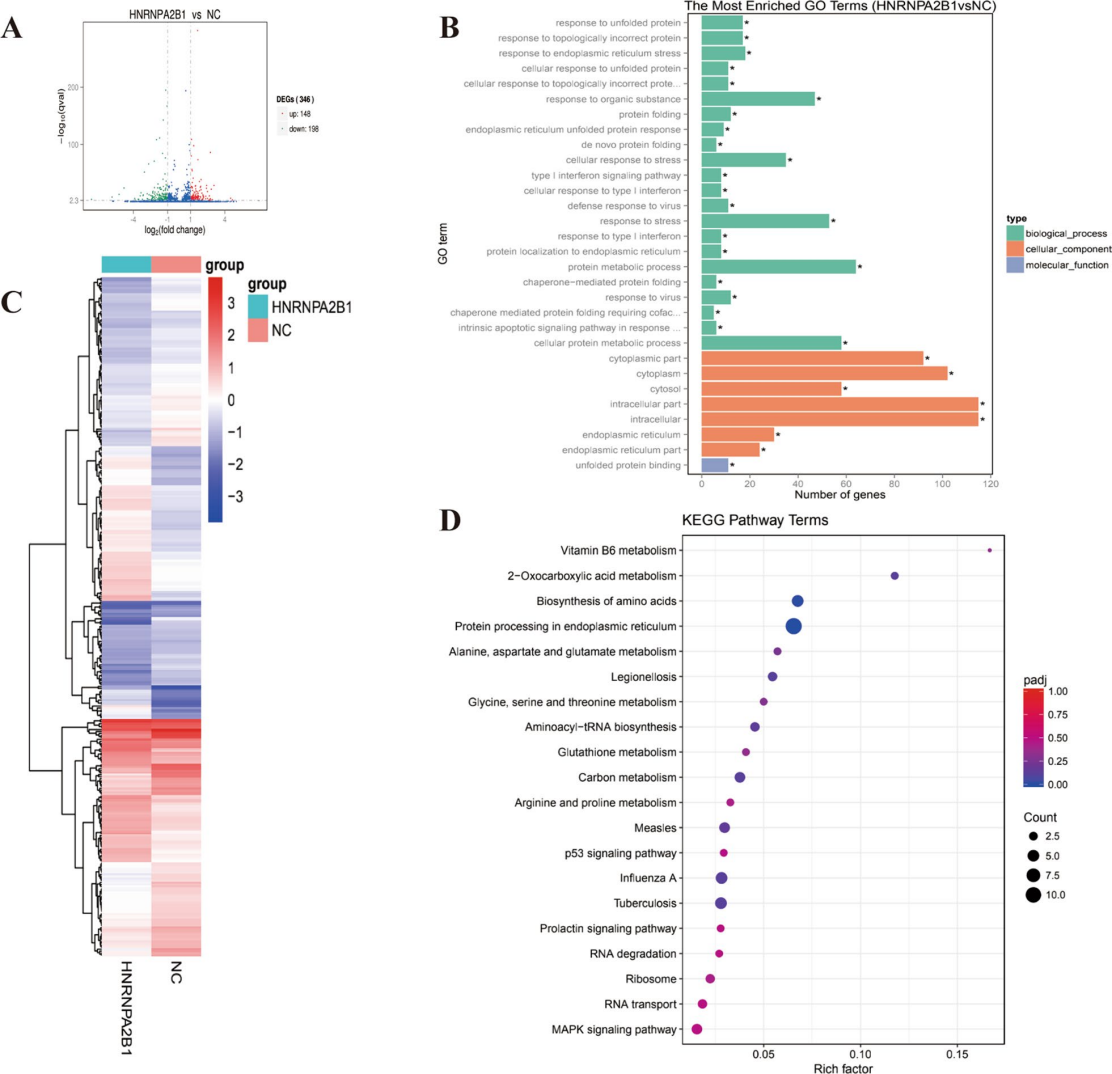
Transcriptome analyses of MM cells transfected with sh-HNRNPA2B1-8226 versus the control sh-NC-8226. **A** Volcano map of the distribution of differentially expressed genes. Up-regulated genes are shown in red, and down-regulated genes in green. **B** Histogram distribution of different genes, showing the pathways with the most significant differences. The green bar represents the biological process pathway, the orange represents the cellular component pathway, and the blue indicates the molecular function pathway. **C** Heatmap of different genes clustered in sh-HNRNPA2B1-8226 and sh-NC-8226. The color from blue to red indicates that log10 (FPKM + 1) is from low to high. **D** KEGG pathway enrichment results for differentially expressed genes between MM cells transfected with sh-HNRNPA2B1-8226 and control sh-NC-8226

To analyze the role of HNRNPA2B1 in myeloma cells, we compared the transcriptome sequencing results for MM cells transfected with sh-HNRNPA2B1-8226 and the control sh-NC-8226. The analysis identified 346 differentially expressed genes (Additional file 2: S1), of which 148 were up-regulated and 198 were down-regulated (Fig. 3A). Gene Ontology (GO) enrichment showed that the effect of HNRNPA2B1 in myeloma cells is mainly concentrated in the two processes of biological process and cell composition, and that HNRNPA2B1 participates in the regulation of myeloma cells (Fig. 3B). FPKM based on gene read count was used to quantify the differences among the two groups (sh-HNRNPA2B1-8226 and the control sh-NC-8226) for transcriptomics analysis. In the overall FPKM hierarchical clustering diagram, the Log10 (FPKM + 1) value is normalized to scale number and clustered. Genes expressed at high levels are indicated by red, and genes expressed at low levels are indicated by blue. The color from red to blue indicates that log10 (FPKM + 1) is from high to low. As shown in the heatmap, HNRNPA2B1 regulated multiple gene clusters in MM (Fig. 3C). Kyoto Encyclopedia of Genes and Genomes (KEGG) pathway enrichment analysis showed the differential gene enrichment was related to a variety of signaling pathways including proteins processing in endoplasmic reticulum (ER) stress and hypoxia induction, suggesting that HNRNPA2B1 plays an important role in ER stress in the regulation of myeloma (Fig. 3D).

**Fig. 4:**
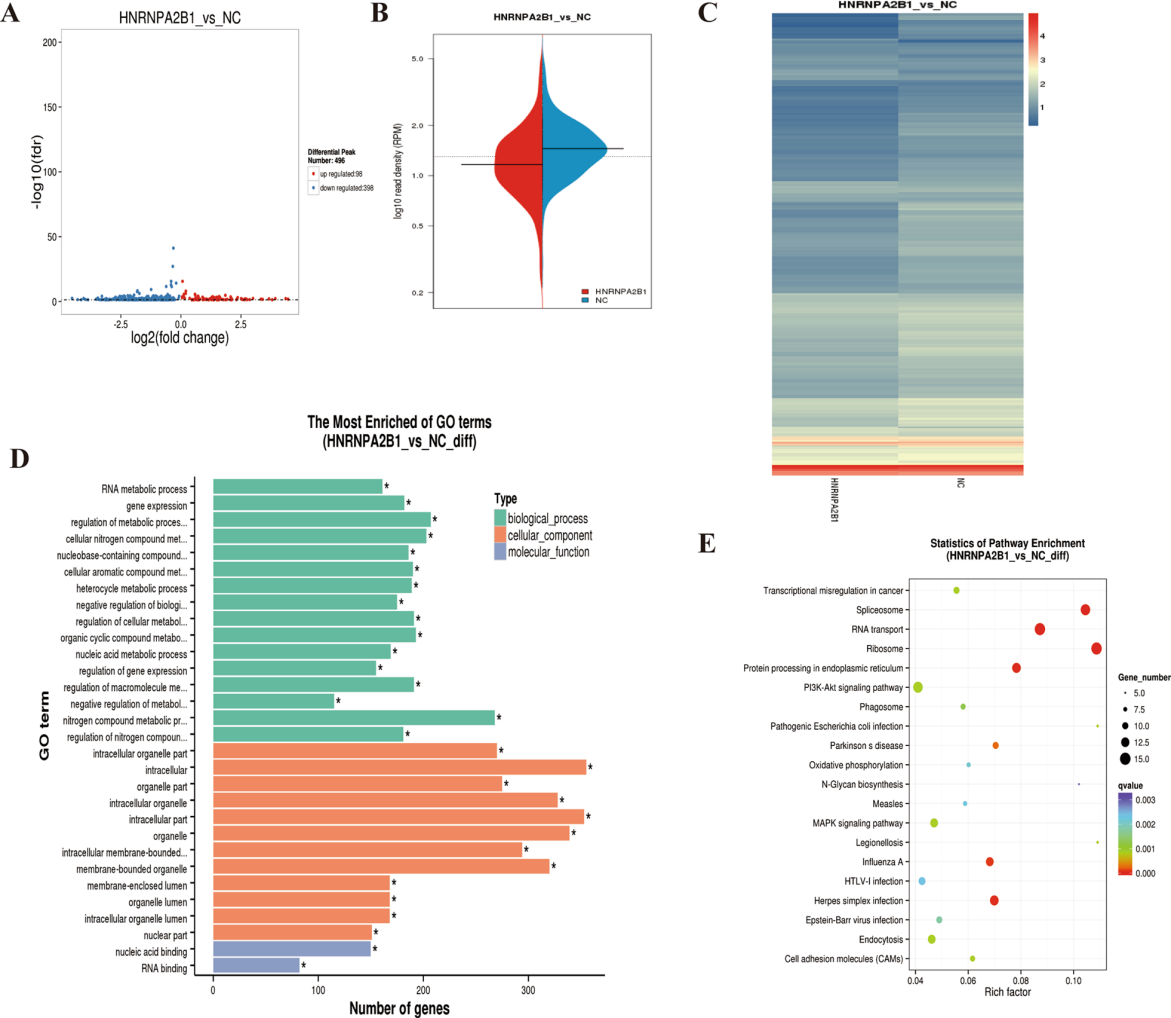
M6A methylation immunoprecipitation sequencing analyses of MM cells transfected with sh-HNRNPA2B1-8226 versus the control sh-NC-8226. **A** Volcano map of the distribution of differentially expressed genes. Up-regulated genes are shown in red, and down-regulated genes in blue. **B** Violin plot of the distribution of RPM in the difference peak. **C** Heatmap of differential peak signal distribution in sh-HNRNPA2B1-8226 and sh-NC-8226. **D**, **E** GO pathway enrichment (**D**) and KEGG pathway enrichment (**E**) results for differentially expressed peaks between MM cells transfected with sh-HNRNPA2B1-8226 and control sh-NC-8226

To analyze how HNRNPA2B1 regulates m6A methylation in myeloma cells, m6A methylation immunoprecipitation sequencing (MeRIP) was performed in MM cells transfected with sh-HNRNPA2B1-8226 and the control sh-NC-8226. The results showed that 496 peaks were differentially expressed in MeRIP (Additional file 3: S2), of which 98 were up-regulated and 398 were down-regulated (Fig. 4A). The volcano plot indicated the distribution of RPM based on the difference peak between the two groups. The black line represents the median value in each group of experiments. The dotted line represents the average value of the two groups (Fig. 4B). The heatmap of differential peak signal distribution showed that HNRNPA2B1 regulates the RPM signal (Fig. 4C). GO enrichment indicated that the significantly different peaks were enriched in a variety of regulatory signal pathways of biological processes, cell components, and molecular functions (Fig. 4D). KEGG enrichment analysis showed that the pathways of transcription regulation, ER stress, oxidative phosphorylation, MAPK signaling pathway, EBV infection, and cell adhesion molecules were influenced. These findings suggest that HNRNPA2B1 participates in the regulation of m6A methylation levels of related genes in multiple signaling pathways (Fig. 4E).

### TLR4 is a downstream target of HNRNPA2B1

**Fig. 5:**
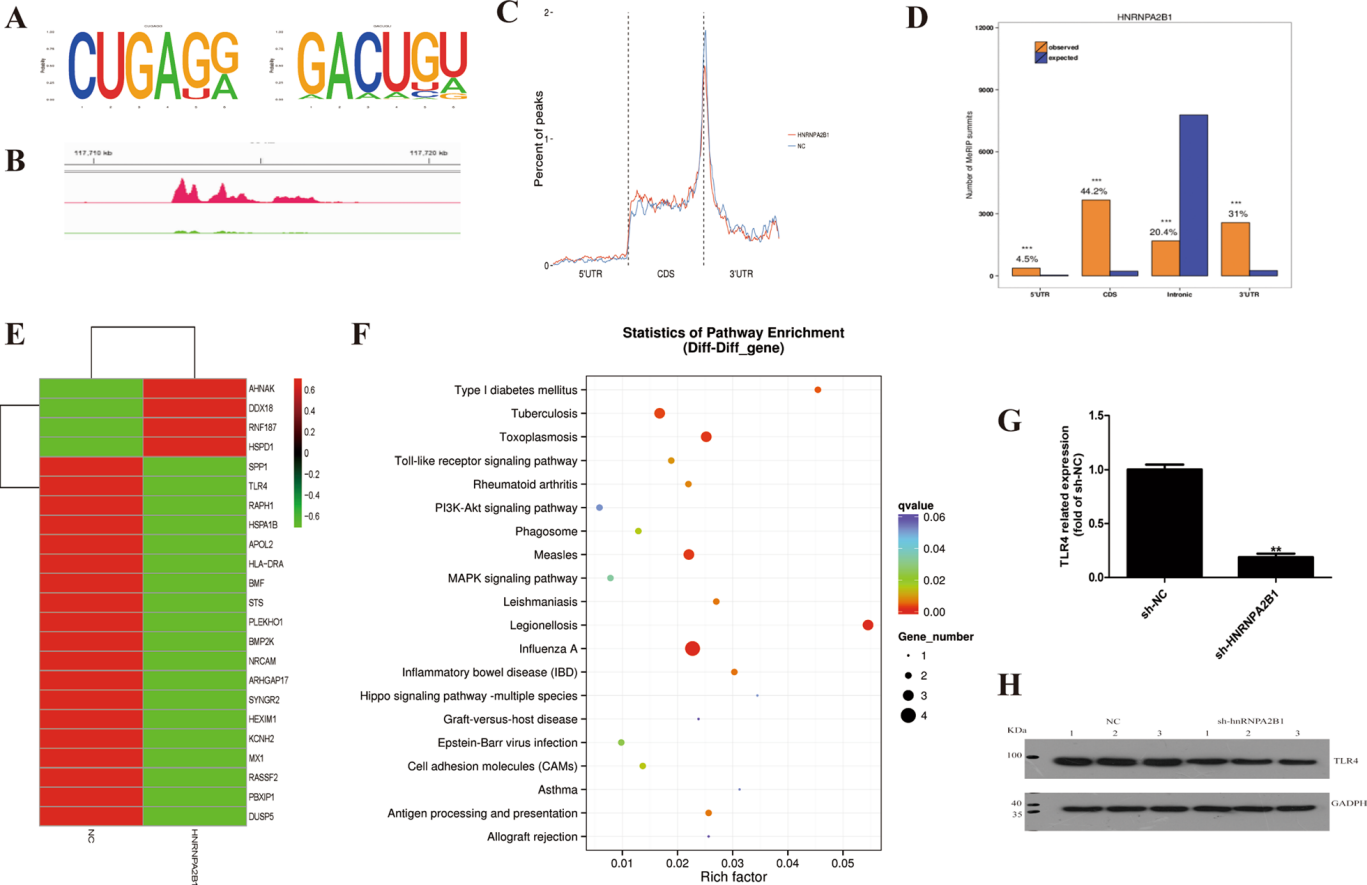
Correlation analysis of HNRNPA2B1 and TLR4 expression in RPMI 8226 cells transfected with sh-HNRNPA2B1-8226 and control sh-NC-8226. **A** Differential motif between RPMI 8226 cells transfected with sh-HNRNPA2B1-8226 and sh-NC-8226. **B** IGV tracks indicated the m6A modification position of the TLR4 gene. **C** The percentage of peak distribution in different regions (5′UTR, CDS and 3′UTR) of sh-HNRNPA2B1-8226 and the control sh-NC-8226. **D** The distribution histogram of peak on four transcriptional functional regions (5′UTR, 3′UTR, CDs and intronic). **E** Heatmap of differential peak between MM cells transfected with sh-HNRNPA2B1-8226 and sh-NC-8226. **F** KEGG pathway enrichment results for differentially expressed genes identified by MeRIP sequencing between cells transfected with sh-HNRNPA2B1-8226 and control sh-NC-8226 in MM. TLR4 was enriched in many pathways of KEGG terms. **G** TLR4 gene expression in HNRNPA2B1 knockdown and control cells as analyzed by qRT-PCR. **H** TLR4 protein expression in HNRNPA2B1 knockdown and control cells as analyzed by western blotting

To analyze how HNRNPA2B1 regulates m6A methylation in myeloma cells, m6A methylation immunoprecipitation sequencing (MeRIP) was performed in MM cells transfected with sh-HNRNPA2B1-8226 and the control sh-NC-8226. The m6A consensus sequence (RRACH) motif was shown in Fig. 5A. An enriched peak was detected around the stop codon region of TLR4 mRNA in control RPMI 8226 cells, and the peak was diminished upon HNRNPA2B1 knockdown (Fig. 5B). The percentage of peak distribution in different regions (5'UTR, CDS and 3'UTR) of sh-HNRNPA2B1-8226 and the control cells were shown in Fig. 5C. The distribution histogram of peak on four transcriptional functional regions (5'UTR, 3'UTR, CDs and intronic) is shown in Fig. 5D. To identify the key target of HNRNPA2B1 in the process of m6A methylation regulation, we analyzed correlations in the results of transcriptome sequencing and MeRIP sequencing. Of these, 23 genes were differentially expressed in both the transcriptome and methylation (Additional file 4: S3), including *PLEKHO1*, *KCNH2*, *DDX18*, *NRCAM, RASSF2*, *STS*, *BMF*, *SYNGR2*, *SPP1*, *AHNAK, APOL2*, *TLR4*, *DUSP5*, *BMP2K*, *ARHGAP17*, *HSPD1, MX1, PBXIP1, RNF187*, *RAPH1*, *HEXIM1*, *HLA-DRA* and *HSPA1B* (Fig. 5E). Importantly, TLR4 was identified as a direct target of m6A methylation in the result of our m6A-Seq analysis. In addition, studies have confirmed that TLR4 participates in the processes of activation of multiple inflammatory pathways and tumorigenesis [15]. Moreover, TLR4 was shown to be associated with the myeloma tumor microenvironment and bortezomib resistance [16]. Therefore, we selected TLR4 as a candidate target of HNRNPA2B1-mediated m6A modification for further investigation.

Enrichment of TLR4 in the GO differential gene pathways was analyzed. TLR4 was enriched in a variety of important signaling pathways, such as alkylation metabolism, cell metabolism regulation, cell negative regulation metabolism, cell cycle complex metabolism regulation, etc. KEGG enrichment analysis showed that the pathways of transcription regulation, ER stress, oxidative phosphorylation, MAPK signaling pathway, EBV infection, and cell adhesion molecules were influenced (Fig. 5F). These findings suggest that HNRNPA2B1 participates in the regulation of m6A methylation levels of related genes in multiple signaling pathways.

From the KEGG differential gene pathway analysis, TLR4 enrichment was also involved in a variety of signaling pathways, such as the PI3K-Akt pathway, influenza virus A pathway, hepatitis B signaling pathway, phagocytic signaling pathway, Toll-like receptor signaling pathway, *Salmonella* infection signaling pathway, cancer proteoglycan, hypoxia inducible factor signaling pathway, inflammatory bowel disease signaling pathway, rheumatoid arthritis signaling pathway, ER stress, nuclear factor (NF)-κB signaling pathway, and many other important signaling pathways related to tumors and inflammation.

Next, qRT-PCR and western blot analyses were conducted to verify the relationship between TLR4 and HNRNPA2B1. We observed that knockdown of HNRNPA2B1 suppressed the mRNA and protein expression levels of TLR4 (Fig. 5G and H). Together, our data suggested that HNRNPA2B1 promotes tumor progression of MM through the regulation of TLR4 expression.

### HNRNPA2B1 and TLR4 expression is associated with the survival of MM patients in NCBI GEO microarray datasets

**Fig. 6:**
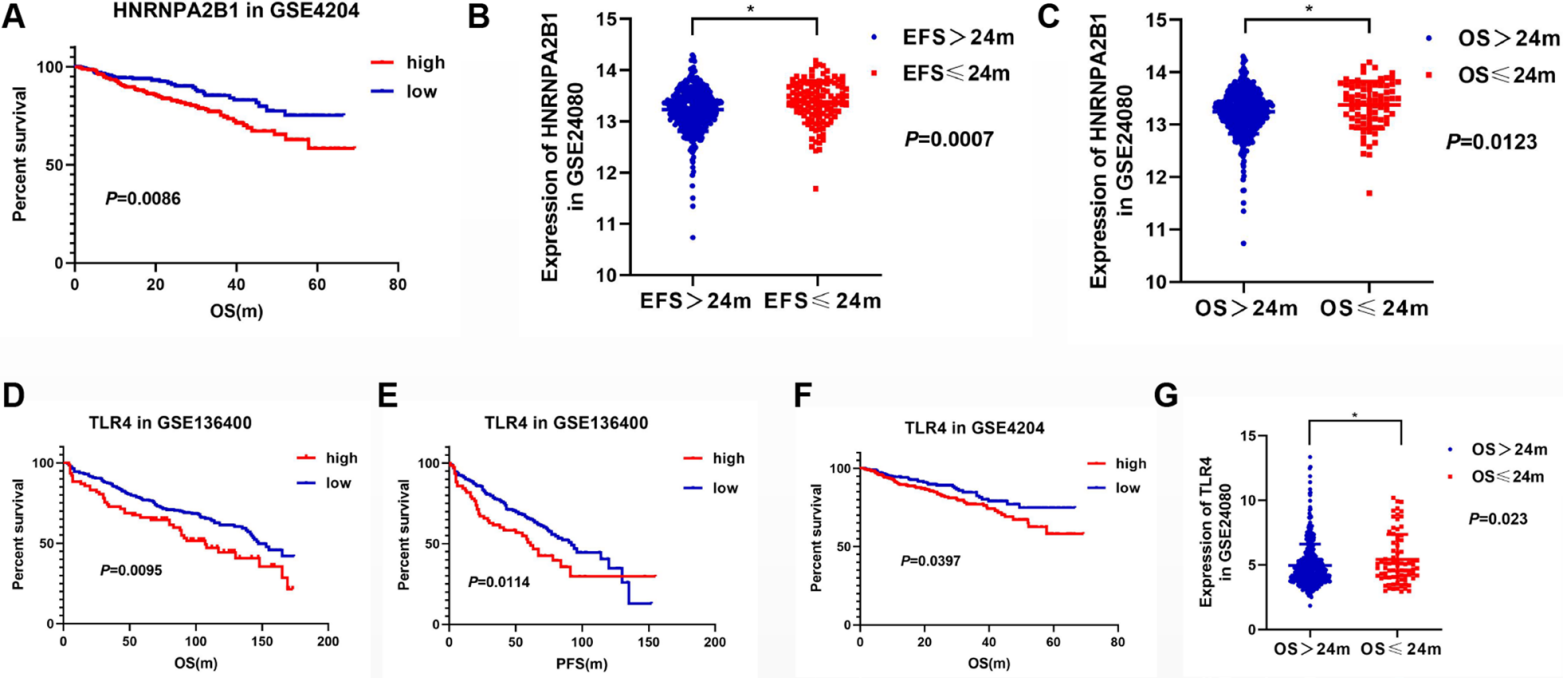
Survival analysis based on the expression levels of HNRNPA2B1 and TLR4 in GEO datasets of MM patients. **A** The correlation between HNRNPA2B1 expression and the OS of MM patients in GSE4204 dataset was conducted by Kaplan–Meier analysis. **B**, **C** Expression of RNPA2B1 in different survival subgroups (EFS_24m_ and OS_24m_) in GSE24080. **D**, **E** The correlation between TLR4 expression and OS/PFS of MM patients in GSE136400 was conducted by Kaplan–Meier analysis. **F** The correlation between TLR4 expression and OS of MM patients in GSE4204 was conducted by Kaplan–Meier analysis. **G** Expression of TLR4 in different survival subgroups (OS_24m_) in GSE24080. The median values were used as the cut-offs for discrimination of high/low HNRNPA2B1/TLR4 expression

Further, we used datasets to further explore the role of HNRNPA2B1 and TLR4 expression in clinical specimens of MM. Three independent NCBI GEO datasets were applied to validate the effect of HNRNPA2B1 and TLR4 expression on the survival of MM patients. We found that the OS of the HNRNPA2B1^high^ subgroup was significantly poorer than that of the HNRNPA2B1^low^ subgroup in GSE4204 (Fig. 6A, *P* = 0.0086). In GSE24080 set, the subgroups with EFS > 24 months and OS > 24 months showed lower expression of HNRNPA2B1compared to the subgroups with EFS ≤ 24 months and OS ≤ 24 months, (Fig. 6B and C, *P* = 0.0007, 0.0123, respectively), which was consistent with the results from GSE4204 and indicates that MM patients with higher HNRNPA2B1 expression had relatively decreased survival times.

With regard to TLR4 expression in MM patients, we found that the TLR4^high^ subgroup had significantly poorer survival, including both OS and PFS, compared to the TLR4^low^ subgroup in two independent datasets of GSE136400 (Fig. 6D and E, *P* = 0.0095, 0.0114, respectively) and GSE4204 (Fig. 6F, *P* = 0.0397). In GSE24080, TLR4 expression was higher in the OS ≤ 24 months subgroup than in the OS > 24 months subgroup (Fig. 6G, *P* = 0.023). These results identify TLR4 acted as an adverse prognostic factor of survival among MM patients. Moreover, the consistency rate of the HNRNPA2B1/TLR4 expression was about 46% in the GSE4204 dataset, with 123 patients (22.8%) having both high HNRNPA2B1 and TLR4 expression, and 125 patients (23.2%) having both low HNRNPA2B1 and TLR4 expression. Altogether, the above results indicate a possible role of HNRNPA2B1 in the positive regulation of TLR4 in MM patient through m6A methylation.

## Discussion

M6A is the most common RNA chemical modification in human mRNA. Recently, with the advancement of high-throughput sequencing and antibody-based technology, researchers have been able to accurately map the site of m6A and further study its biological functions [17]. Although studies have shown potential functions of m6A in regulating mRNA decay, translation, and processing, the pathologic significance of m6A in MM has remained unknown. In the present study, we found that m6A levels were significantly elevated in MM due to upregulation of the methyl reader HNRNPA2B1. Our observations in MM cells with HNRNPA2B1 knockdown indicated that HNRNPA2B1 plays a critical role in promoting MM proliferation and inhibiting MM apoptosis. In addition, we found that TLR4 is a downstream target of m6A modification mediated by HNRNPA2B1. In summary, our results suggest that HNRNPA2B1 and its associated m6A modification promote MM progression by regulating the expression of the key gene TLR4 at the post-transcriptional level. Therefore, our findings reveal a new epigenetic regulatory mechanism contributing to the progression of MM.

HNRNPA2B1 participates in multiple biological processes in many diseases, especially cancers. One study demonstrated that down-regulation of HNRNPA2B1 can inhibit the cell proliferation, tumor invasion, and cell cycle progression through the PI3K/AKT signaling pathway of cervical cancer cells, thus triggering cell apoptosis [18]. HNRNPA2B1 also was identified as an oncogene in head and neck cancer, which promotes epithelial to mesenchymal transition through the AKT/PKB signaling pathway [19]. Additionally, the protein level of HNRNPA2B1 can be modulated by its ubiquitination status through miR503HG, thereby regulating HCC metastasis and migration in hepatocellular carcinoma (HCC) [20]. Recently, the role of HNRNPA2B1 in MM was also explored by Jiang and colleagues, who found that HNRNPA2B1 expression is a negative prognostic factor in MM. Mechanistically, interleukin enhancer binding factor 3 (ILF3) was identified as an m6A target site of HNRNPA2B1 [21]. In the present study, we identified TLR4 as the HNRNPA2B1 target site. Notably, we did not observe ILF3 among the core genes identified by RNA-sequencing and MeRIP sequencing. These inconsistent results may be due to different experiment conditions, as well as slight differences in the sequencing technologies employed.

TLR4, as a pattern recognition receptor, mainly mediates endogenous immunity and immune presentation, regulating the inflammatory response and participating in the expression of inflammatory factors. Recent studies have suggested that TLR4 expression correlates with cancer progression and TLR4 plays a key role in disrupting tumor cell apoptosis regulation, decreasing tumor cell apoptosis, and promoting tumor cell proliferation. For example, TLR4 activation releases immunosuppressive exosomes, promotes tumor progression, and accelerates the metastatic process [22]. In MM, TLR4 expression was increased in the bone marrow cells of MM patients compared to healthy volunteers. Flow cytometric analyses also indicated upregulation of TLR4 in MM cells [23]. Mechanistically, TLR4 suppresses ER stress-related apoptosis and promotes MM cell proliferation and survival through the PERK-CHOP pathway [24]. A recent study highlighted the overexpression of TLR4 according to disease stage and implied that TLR4 inhibition reduced mesenchymal stromal cell activity and decreased MM cell growth [25]. Consistent with these previous studies, our results also imply that a high level of TLR4 expression correlates with poor prognosis in MM patients.

m6A methylation and its regulators links epigenetic transcriptomics to tumor development. Studies have shown that m6A modification plays critical and diverse roles in the progression of various cancers [26]. m6A methylation and its enzyme METTL3 are upregulated in glioblastoma stem cells and HCC, and the high expression of METTL3 induces SOCS2 degradation by m6A methylation, thus promoting tumor cell growth and survival [27]. In another study, fat mass and obesity-associated protein (FTO), an m6A eraser, showed altered expression in intrahepatic cholangiocarcinoma, and low FTO expression predicted poor prognosis [28]. These studies imply oncogenic functions of m6A methylation. On the other hand, several studies have shown that m6A may work as a tumor suppressor in some cancers. For example, one study found that METTL4 down-regulation led to m6A modification dysregulation, which was related to tumor metastasis and poor prognosis in HCC [29]. In our study, we suggested that m6A modification has an oncogenic function in MM. Dysregulation of the m6A level seems to act as a “double-edged sword” in cancer progression, and further studies are needed to elucidate the key role of m6A.

The findings of this study must be considered in light of some limitations. According to the results in Fig. 1B, HNRNPA2B1 and eukaryotic translation initiation factor 3 (eIF3, B, D, E, K) were both overexpressed genes. eIF3 is the most complex eukaryotic translation initiation factor and is crucial for tumorigenesis [30–32]. eIF3 regulates cap-dependent translation processes and also plays an important role in cap-dependent translation regulation by binding to putative internal ribosome entry sites [33, 34]. The role of eIF3 in MM is not fully understood, and this can be a future direction of research. eIF3 may also represent a potential mechanism for the regulation of TLR4 in MM. However, our study did not include experiments to exclude it. Further exploration of the role of eIF3 in MM is needed in the future.

In summary, the results of the present study indicate that HNRNPA2B1 acts as oncogene in MM development. Our experiments suggested the HNRNPA2B1 promotes MM proliferation and inhibits apoptosis through the epigenetic regulation of TLR4 by m6A modification. Moreover, HNRNPA2B1 expression was significantly increased in MM and shown to correlate with poor prognosis in MM patients. Altogether, our results suggest HNRNPA2B1 as a potential therapeutic target for MM.

## Supplementary Information

**Additional file 1: Table S1.** Primers used for qRT-PCR.

**Additional file 2. **Transcriptome sequencing identified 346 differentially expressed genes for MM cells transfected with sh-HNRNPA2B1-8226 and the control sh-NC-8226.

**Additional file 3. **MeRIP sequencing identified 496 differentially peaks for MM cells transfected with sh-HNRNPA2B1-8226 and the control sh-NC-8226.

**Additional file 4. **The 23 genes differentially expressed in both the transcriptome and methylation sequencing for MM cells transfected with sh-HNRNPA2B1-8226 and the control sh-NC-8226.

## Data Availability

The data analyzed during this study are included in the published article and supplementary materials. No more additional data is generated.

## Abbreviations

DEGs: Differentially expressed genes
EFS: Event-free survival
ER: Endoplasmic reticulum
FTO: Fat mass and obesity-associated protein
GEO: Gene expression omnibus
HCC: Hepatocellular carcinoma
HNRNPA2B1: Heterogeneous nuclear Ribonucleoprotein A2B1
ILF3: Interleukin enhancer binding factor 3
KEGG: Kyoto Encyclopedia of Genes and Genomes
MeRIP: Methylation immunoprecipitation sequencing
MM: Multiple Myeloma
NC: Normal control
NF-κB: Nuclear factor -κB
OS: Overall survival
PI: Propidium iodide
TLR4: Toll-like receptor 4
