# Correction: HNRNPA2B1-mediated m6A modification of TLR4 mRNA promotes progression of multiple myeloma

**DOI:** 10.1186/s12967-023-03904-2

**Published:** 2023-01-30

**Authors:** Chuiming Jia, Yiwei Guo, Yao Chen, Xinya Wang, Qiuting Xu, Yu Zhang, Lina Quan

**Affiliations:** 1 Hematology Department, Harbin Medical University Cancer Hospital, Harbin, Heilongjiang People’s Republic of China; 2 Immunology Department, Harbin Medical University, Harbin, Heilongjiang People’s Republic of China

**Correction** **:** **Journal of Translational Medicine (2022) 20:537** 10.1186/s12967-022-03750-8

Following publication of the original article [1], we have been notified that authors’ sequence and affiliations were published incorrectly. This should be as follows:

Chuiming Jia^1^, Yiwei Guo^1^, Yao Chen^1^, Xinya Wang^1^, Qiuting Xu^1^, and Yu Zhang^2^, Lina Quan^1^*
