# Supplementary material for: HNRNPA2B1-mediated m6A modification of TLR4 mRNA promotes progression of multiple myeloma
Source: J Transl Med. 2022 Nov 18;20:537. doi: 10.1186/s12967-022-03750-8 (PMC9673362; doi:10.1186/s12967-022-03750-8)
Supplement: Supplementary file 1 — Additional file 1: Table S1. Primers used for qRT-PCR. [file 12967_2022_3750_MOESM1_ESM.docx]

Table S1. Primers used for qRT-PCR.

| Gene | Direction | Primer |
| --- | --- | --- |
| *METTL3* | F | GAGTGCATGAAAGCCAGTGA |
|  | R | ACTGGAATCACCTCCGACAC |
| *METTL14* | F | AAAATGCTGGACTTGGGATG |
|  | R | CCATGAGGCAGTGTTCCTTT |
| *WTAP* | F | GCGACTAGCAACCAAGGAAC |
|  | R | CATTTTGGGCTTGTTCCAGT |
| *FTO* | F | ACCCCTTCACCAAGGAGACT |
|  | R | AAAACTGCAGGCTCAAAGGA |
| *ALKBH5* | F | TTCAAGCCTATTCGGGTGTC |
|  | R | CGGGGTGCATCTAATCTTGT |
| *YTHDF1* | F | CCCGTCTACCTGCTCTTCAG |
|  | R | TGACCGGTTTGTTGTCGTTA |
| *YTHDF2* | F | AGCCCCACTTCCTACCAGAT |
|  | R | TCATTGGCAAAAGCTGACTG |
| *YTHDF3* | F | CGTGCTGGAAAAGCTAAAGG |
|  | R | AATGTCCACTGCCATTCACA |
| *eIF3A* | F | GATCGAGAGGATCGCTTCAG |
|  | R | CATCAGCACGTCTCCAAGAA |
| *eIF3B* | F | GCCTCCTGCAGAAGAACAAC |
|  | R | CTTCCGGAAATCTTCCATCA |
| *eIF3D* | F | CACGGAGCTGAAGAACAACA |
|  | R | GTCAATGACGCAGCGTAAAA |
| *eIF3E* | F | CAACCAGGGATGGTAGGATG |
|  | R | TGCATCCCAATTCTGCATTA |
| *eIF3F* | F | CTGCTTTAGCCCCAACAGAG |
|  | R | TTGCTGTTGAGCATGGTCTC |
| *eIF3G* | F | GTTCAAGATTGTCCGCACCT |
|  | R | GCAGTTCAGGTCCTCTTTGC |
| *eIF3K* | F | GCCAAGGAAAATGCCTATGA |
|  | R | ATTGGCCGTTCTTCTTGATG |
| *IGF2BP1* | F | ATCCGCAACATCACAAAACA |
|  | R | TTATGGGCCAGGATCTTCAG |
| *IGF2BP2* | F | CCTGTGCCAGTGCTGAGATA |
|  | R | AAACTGGTGATGGGGGTACA |
| *IGF2BP3* | F | AGTTGTTGTCCCTCGTGACC |
|  | R | GTCCACTTTGCAGAGCCTTC |
| *hnRNPA2B1* | F | GGCTACGGAGGTGGTTATGA |
|  | R | ATAACCCCCACTTCCTCCAC |
| *GAPDH* | F | TGTGGGCATCAATGGATTTGG |
|  | R | ACACCATGTATTCCGGGTCAAT |
